# Induction of neutralizing antibodies against tier 2 human immunodeficiency virus 1 in rhesus macaques infected with tier 1B simian/human immunodeficiency virus

**DOI:** 10.1007/s00705-019-04173-5

**Published:** 2019-02-28

**Authors:** Ai Himeno, Yuki Ishida, Hiromi Mori, Kanako Matsuura, Minako Kikukawa, Hiromi Sakawaki, Tomoyuki Miura

**Affiliations:** Laboratory of Primate Model, Research Center for Infectious Diseases, Institute for Frontier Life and Medical Science, Kyoto University, 53 Shogoin kawahara-cho, Sakyo-ku, Kyoto, 606-8507 Japan

## Abstract

We previously developed CCR5-tropic neutralization-resistant simian/human immunodeficiency virus (SHIV) strains and a rhesus macaque model of infection with these SHIVs. We induced the production of neutralizing antibodies (nAbs) against HIV-1 by infecting rhesus macaques with different neutralization-resistant SHIV strains. First, SHIV-MK1 (MK1) (neutralization susceptible, tier 1B) with CCR5 tropism was generated from SHIV-KS661 using CXCR4 as the main co-receptor. nAbs against parental-lineage and heterologous tier 2 viruses were induced by tier 1B virus (MK1) infection of the rhesus macaque MM482. We analyzed viral resistance to neutralization over time in MM482 and observed that the infecting virus mutated from tier 1B to tier 2 at 36 weeks postinfection (wpi). In addition, an analysis of mutations showed that N169D, K187E, S190N, S239, T459N (T459D at 91 wpi), and V842A mutations were present after 36 wpi. This led to the appearance of neutralization-resistant viral clones. In addition, MK1 was passaged in three rhesus macaques to generate neutralization-resistant SHIV-MK38 (MK38) (tier 2). We evaluated nAb production by rhesus macaques infected with SHIV-MK38 #818 (#818) (tier 2), a molecular clone of MK38. Neutralization of the parental lineage was induced earlier than in macaques infected with tier 1B virus, and neutralization activity against heterologous tier 2 virus was beginning to develop. Therefore, CCR5-tropic neutralization-resistant SHIV-infected rhesus macaques may be useful models of anti-HIV-1 nAb production and will facilitate the development of a vaccine that elicits nAbs against HIV-1.

**Electronic supplementary material:**

The online version of this article (10.1007/s00705-019-04173-5) contains supplementary material, which is available to authorized users.

## Introduction

Antiretroviral agents are used against human immunodeficiency virus type 1 (HIV-1), but eliminating latent HIV-1 is difficult [1–9]. Therefore, suppression and prevention of HIV-1 infection by passive administration of neutralizing antibodies (nAbs) and induction of nAbs by vaccination would be beneficial [10–17]. Few HIV-1-infected patients (10–30%) produce nAbs, and about 1% of infected people generate highly potent nAbs with broad neutralization coverage of HIV (elite neutralizers) [18, 19]. Due to advances in antigen-specific B-cell isolation techniques, broadly neutralizing monoclonal antibodies have been isolated from HIV-1-infected patients [20–23]. Passive administration of these nAbs was protective against simian/human immunodeficiency virus (SHIV) in a macaque model [24–30]. However, inducing potent and broadly reactive nAbs by vaccination is problematic. Although the production of potent nAbs with broad cross-reactivity is related to somatic hypermutation [31–34], the mechanism of induction is unknown. An animal model in which nAbs are produced would facilitate clarification of the mechanism of induction of nAbs against HIV-1, as well as the development of effective vaccines.

The rhesus macaque model of simian immunodeficiency virus (SIV) infection is important as an animal model of AIDS for pathogenicity studies and vaccine development. However, the envelope protein (Env) of SIV has a low level of amino acid sequence similarity to that of HIV-1 [35], and nAbs against the two viruses are not cross-reactive [36]. By contrast, SHIV [37], which is SIV containing the *env* gene of HIV-1, can be used to evaluate nAbs against the Env protein of HIV-1.

Controlling HIV and SIV is difficult, as they use CCR5 as a co-receptor; however, SHIV-89.6P (CXCR4) is easy to control [38]. Seaman *et al*. [39] reported that clustering analysis of the patterns of sensitivity defined four subgroups of clinical HIV-1 strains: those having very high (tier 1A), above-average (tier 1B), moderate (tier 2), or low (tier 3) sensitivity to antibody-mediated neutralization, with the majority of viruses belonging to tier 2.　Indeed, the production of antibodies in rhesus macaques suppressed replication of SHIV-KS661(KS661) (CXCR4-tropic, tier 1B) [40]. SHIV-SF162P3 and SHIV-AD8 (tier 2) are used as challenge viruses in vaccine development [33, 41–46]. We generated several different tier 2 challenge SHIVs to increase the reliability of the research. SHIV-89.6 is frequently used in vaccine studies [47–50] and thus was selected for this study. First, KS661 (SHIV-89.6 strain), which mainly uses CXCR4 as a co-receptor, was modified to produce SHIV-MK1 (MK1) (tier 1B) and inoculated into rhesus macaques. Next, viruses from the infected macaques were passaged in two macaques, resulting in neutralization-resistant SHIV-MK38 (MK38) (tier 2) [51]. Ishida *et al.* [52] produced the MK38 molecular clone SHIV-MK38 #818 (#818) (tier 2).

In this study, we evaluated nAb production by rhesus macaques infected with CCR5-tropic tier 1 and tier 2 SHIV. nAbs against tier 2 virus were induced by tier 1B virus infection, and production of nAbs against tier 2 virus began earlier in Tier 2 virus infection. Our findings provide important insights that might be applicable to HIV-1 vaccine development.

## Materials and methods

### Cell culture

HEK293T (293T) cells were cultured in Dulbecco’s modified Eagle’s medium (DMEM) (Fujifilm Wako Pure Chemical Corporation, Osaka, Japan) supplemented with 10% (vol/vol) heat-inactivated fetal bovine serum (FBS; JR Scientific Inc., Woodland, CA, USA). TZM-bl cells were cultured in DMEM supplemented with 10% (vol/vol) heat-inactivated FBS, 2 mM sodium pyruvate (MP Biomedicals Inc., Santa Ana, CA, USA) and 4 mM L-glutamine (Fujifilm Wako Pure Chemical Corporation). Cells were harvested and passaged using trypsin/ethylenediaminetetraacetic acid solution (Nacalai Tesque, Kyoto, Japan) and were maintained at 37 °C in a humidified atmosphere containing 5% CO_2_.

### Viruses and animal experiments

SHIV-MK1, SHIV-MK1-first passage, SHIV-MK1-second passage, and SHIV-MK38 were described previously [51], as was SHIV-MK38#818 [52]. Based on the sequence information about co-receptor tropism of HIV-1 [53, 54], we designed neutralization-susceptible CCR5-tropic (tier 1B) MK1 by introducing five amino acid mutations (E305K, R306S, R318T, R319G, and N320D). We inoculated MK1 intravenously into two rhesus macaques (MM482 and MM483). To allow MK1 to adapt, we conducted *in vivo* passages from macaque M482 to macaque MM498 (SHIV-MK1-first passage), and subsequently to macaque MM504 (SHIV-MK1-second passage). This enhanced viral replication and the re-isolated virus was designated SHIV-MK38 (MK38). Next, we inoculated MK38 intravenously into rhesus macaques (MM481, MM501, and MM502) [51]. The molecular clone SHIV-MK38#818 (#818) (tier 2) was produced by Ishida et al. [52]. We mimicked the infection route of HIV-1 to humans and inoculated #818 into the rectum of rhesus macaques (MM 596, MM 597, and MM 599; Table 1) [52]. R5 virus infects intestinal memory CD4-positive T cells [55, 56]. Indian-origin rhesus macaques were used in accordance with the institutional regulations of the Committee for Experimental Use of Non-human Primates of the Institute for Frontier Life and Medical Sciences, Kyoto University, Kyoto, Japan. Macaques were housed in a biosafety level 3 facility and all procedures were performed in this facility.

**Table 1:** Plasma analyzed for neutralization activity (Matsuda *et al*., 2010, Virology. Ishida *et al*., 2015, JGV)

Macaque ID	Virus		Infected pathology				Reference
	Strain	Tier	Methods	Peak of Plasma Viral Load (copies/mL)	Set point of Plasma Viral Load (copies/mL)	CD4 count in Peripheral Blood	
*MM482	MK1 (molecular clone)	1B	20000TCID50/Intra Venous	10^7^-10^8^	10^3^-10^4^	transiently decreased and recovered	Matsuda et al., 2010, Virology.
MM483				10^6^	undetectable	Not Available	
*MM498	MK1-1stPassage	1B-2	Intra Venous	10^7^-10^8^	10^4^	Not Available	
*MM504	MK1-2ndPassage	1B-2	Intra Venous		10^5^-10^6^	continuous reduction without signs of recovery	
*MM481	MK38	2	20000TCID50/Intra Venous		10^6^-10^7^		
*MM501					10^4^		
*MM502					10^5^		
MM596	#818 (molecular clone)		10000TCID50/Intra Rectal		undetectable	reduction	Ishida et al., 2015, JGV.
*MM597					10^5^-10^6^		
MM599					undetectable		

* : Persistently infected macaque

### Pseudotype viruses

Pseudotype viruses harboring the *env* gene of MK1, #818, murine leukemia virus (MLV), or clade B panel viruses (NIH AIDS reagent program) were prepared by co-transfecting 293T cells with pSGΔenv and pcDNA3.1 vectors expressing the respective *env* genes. We obtained pSGΔenv, pcDNA3.1 vectors expressing clade B *env*, and vectors expressing MLV *env* from the NIH AIDS reagent program. To construct the pcDNA3.1 vector expressing the *rev* and *env* genes of MK1 and #818, approximately 3.0 kb of the region including the *rev* and *env* genes of pMK1 [51] and pMK38#818 [52] were amplified by PCR using the primers IFrevF (GCCTTAGGCATCTCCTAT) and SHenv7R (GGAGTATTCATATACTGTCCC). PCR was performed as follows: one cycle of denaturation (94 °C for 2 min), 30 cycles of amplification (98 °C for 10 s, 52 °C for 30 s, and 68 °C for 90 s) and a final extension (68 °C for 10 min) using KOD Plus Neo buffer, 0.2 mM dNTPs, 15 µM primers, 0.02 U of KOD Plus NEO (Toyobo Co., Ltd, Osaka, Japan), and a template. Approximately 5.5 kb of the *env*-deleted region from pcDNA3.1-SHIVMNA [57] (pcDNA3.1-SHIV-MNA *env* was generated by InFusion cloning using the pcDNA3.1 vector and SHIV-MNA *env* PCR product) was amplified by PCR using the primers SHenv7F (GGGACAGTATATGAATACTCC) and IFrevR (ATAGGAGATGCCTAAGGC). PCR was performed as follows: 1 cycle of denaturation (94 °C, 2 min), 30 cycles of amplification (98 °C for 10 s, 52 °C for 30 s, and 68 °C for 3 min), and a final extension (68 °C, 10 min). The buffer and polymerase were as above. The PCR products were purified using a NucleoSpin Gel and PCR Clean-up Kit (TaKaRa Bio Inc., Shiga, Japan), and *env*-depleted pcDNA3.1 was reacted with the inserted *env* DNA. Cloning was conducted using an In-Fusion HD Cloning Kit (TaKaRa), and the resulting plasmid DNA was introduced into Stbl3 cells by electroporation.

Pseudotype viruses harboring the *env* gene obtained from the plasma of MM482 at weekly intervals after infection were prepared by co-transfecting 293T cells with pSGΔenv and pcDNA3.1 vectors expressing the respective *env* genes. We produced pSGΔenv in the same manner as above. Viral RNA was extracted from plasma using a QIAamp Viral RNA Mini Kit (QIAGEN, Hilden, Germany) according to the manufacturer’s protocol. cDNA, including the *env* gene, was synthesized from the extracted RNA by reverse transcription using random hexamers (Invitrogen, Waltham, MA, USA) and SuperScript IV Reverse Transcriptase (Invitrogen). To construct the pcDNA3.1 vector expressing the *rev* and *env* genes of viruses obtained from MM482, at weekly intervals after infection, approximately 3.3 kb of the region including the *rev* and *env* genes in the cDNA template was amplified by PCR using the primers SHenv0F (AGAGCAAGAAATGGAGCCAG) and SHenv8.5R (CCATAGCCAGCCAAATGTCT). PCR was performed as follows: 35 cycles of amplification (98 °C for 10 s, 53 °C for 5 s, and 68 °C for 15 s) using KOD One PCR Master Mix (Toyobo), 15 µM primers, and template. Next, approximately 2.9 kb of the region including the *rev* and *env* genes in the PCR product was amplified by nested PCR using the primers InsertF3 (TTCACCGGCTTAGGCATCTCCTATGGCAGGAAGAAGCGGAGA) and InsertR3 (TTGACCACTTGCCCCCCATTTGTCCCTCACAAGAGAGTGAGCT). PCR was performed as above. The PCR products were purified using a NucleoSpin Gel and PCR Clean-up Kit (TaKaRa) and sequenced directly (Macrogen Japan Corp., Tokyo, Japan). Approximately 5.5 kb of the *env*-deleted region from pcDNA3.1-SC422661 (obtained from the National Institutes of Health [NIH, Bethesda, MD, USA] AIDS reagent program) were amplified by PCR using the primers VectorF3 (AATGGGGGGCAAGTGGTCAA) and VectorR3 (AGGAGATGCCTAAGCCGGTGAA). PCR was performed as follows: 35 cycles of amplification; 98 °C for 10 s, 58 °C for 5 s, and 68 °C for 27 s. The buffer and polymerase were as above. The PCR products were purified, and *env*-depleted pcDNA3.1 was reacted with the inserted *env* DNA. Cloning was conducted using an NEBuilder HiFi DNA Assembly Master Mix (NEB Inc., Beverly, MA, USA), and the resulting plasmid DNA was introduced into Stbl3 cells by electroporation.

### Neutralization assays

Neutralization assays were performed using various pseudoviruses with pooled plasma from HIV-1-infected patients (ZeptoMetrix, Buffalo, NY, USA) as a positive control. Luciferase activity was measured in TZM-bl cells [58]. Plasma was collected from the infected macaques [51, 52] and serially diluted, and the 50% inhibitory dilution of the plasma (ID_50_) was determined with the infectivity of wells lacking plasma defined as 100%. A high ID_50_ value thus indicates potent inhibition. Plasma from infected macaques was inactivated at 56 °C for 60 min and centrifuged at 11,000 rpm for 10 min. The pooled plasma of HIV-1-infected individuals and infected macaques was diluted in fourfold steps from 1:50 to 1:204,800 and pre-incubated with virus (100 TCID_50_) at 37 °C for 60 min. Next, 5,000 TZM-bl cells were cultured with the pre-incubated mixture in the presence of 5 mg of DEAE dextran/mL at 37 °C for 48 h. To measure luciferase activity, 50 of µL cell lysate solution (Toyo B-Net, Tokyo, Japan) was added to each well and the plate was agitated for 15 min. An aliquot of 30 µL of lysate was transferred to a Nunc F96 MicroWell white plate (Thermo Fisher Scientific, Waltham, MA, USA), and the luminescent substrate (50 µL) was added to each well. Luciferase activity was calculated with Mikrowin and a TriStar LB 941 reader (Berthold Technologies, Bad Wildbad, Germany). ID_50_ values were calculated as described previously [39].

As an anti-HIV-1-neutralizing monoclonal antibody, we used KD-247 (which recognizes the epitope GPGR in the V3 region of gp120 and was kindly provided by the Chemo-Sero-Therapeutic Research Institute, Japan). KD-247 was diluted fourfold from 20 to 0.005 µg/mL, and IC_50_ values were calculated as previously described [39].

## Results

### Infection of macaques and antibody production

In MM482 and MM483, the plasma viral RNA level peaked at 10^6^–10^8^ copies/mL and was maintained at 10^3^–10^4^ copies/mL in MM482. In MM498, MM504, MM481, MM501, and MM502, the plasma viral RNA level peaked at 10^7^–10^8^ copies/mL and was maintained at 10^4^–10^7^ copies/mL in all of these macaques. In MM596, MM597, and MM599, the plasma viral RNA level peaked at 10^7^–10^8^ copies/mL and was maintained at 10^5^–10^6^ copies/mL only in MM597. Seven of the ten rhesus macaques developed persistent infections. Many HIV-1-infected patients have a persistent infection with neutralization-resistant virus [39]. To develop a rhesus macaque model of anti-HIV-1 nAb production, we evaluated the neutralization activity and plasma of seven persistently SHIV-infected rhesus macaques (Table 1).

### Neutralization against *env* of parental-lineage virus

Neutralization of parental-lineage virus was evaluated by luciferase assay using human pooled plasma (HPP) from HIV-1-infected patients as the positive control and a pseudovirus containing mouse leukemia virus (MLV) *env* as the negative control. Potent neutralization of MK1, equal to or higher than that of HPP (ID_50_, 831), was detected in MM482, MM504, MM501, MM502, and MM597 (ID_50_, 10,200, 1,337, 2,679, 831, and 4,587, respectively). Additionally, neutralization activity against #818 higher than that of HPP (ID_50_, 287) was detected in MM504, MM501 and MM597 (ID_50_, 1,357, 907 and 474) (Table 2). These results imply that nAbs against tier 2 virus are induced in macaques infected with tier 1B virus. Antibodies against MK1 were induced at 6 wpi in MM482 and at 12 wpi in MM501, MM504, and MM597 (Table 3). In contrast, antibodies against #818 were induced at 64 wpi in MM482, 30 wpi in MM504, and at 12 wpi in MM501 and MM597 (Table 3). These results suggest that nAbs against tier 2 virus are induced earlier in macaques infected with tier 2 virus than in those infected with tier 1B virus. Furthermore, the neutralization activity increased over time.

**Table 2:**
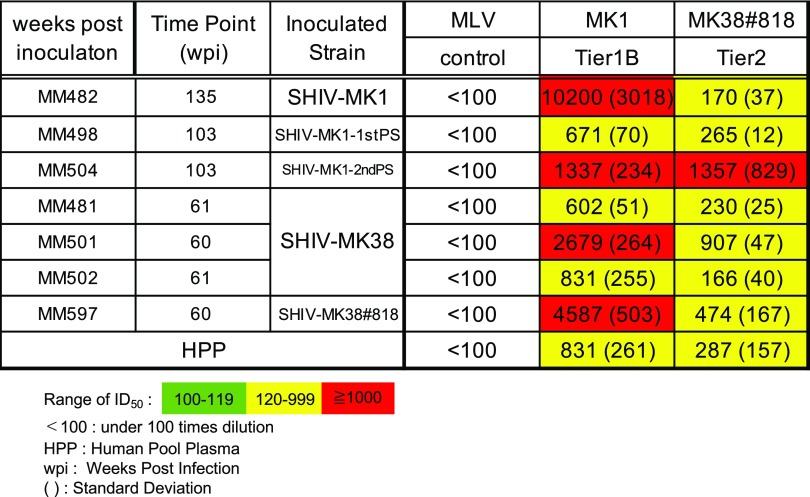
Neutralization activity against parental-lineage virus

**Table 3:**
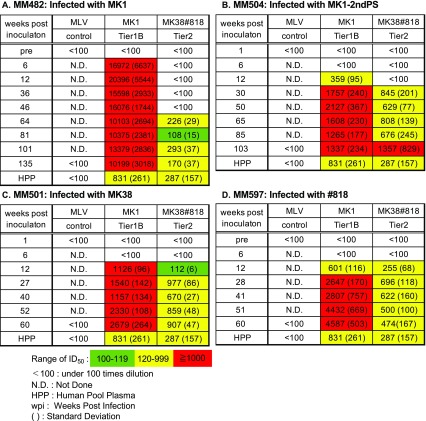
Neutralization activity and breadth against parental-lineage virus over time

### Neutralization against the ENV protein of heterologous viruses

**Fig. 1:**
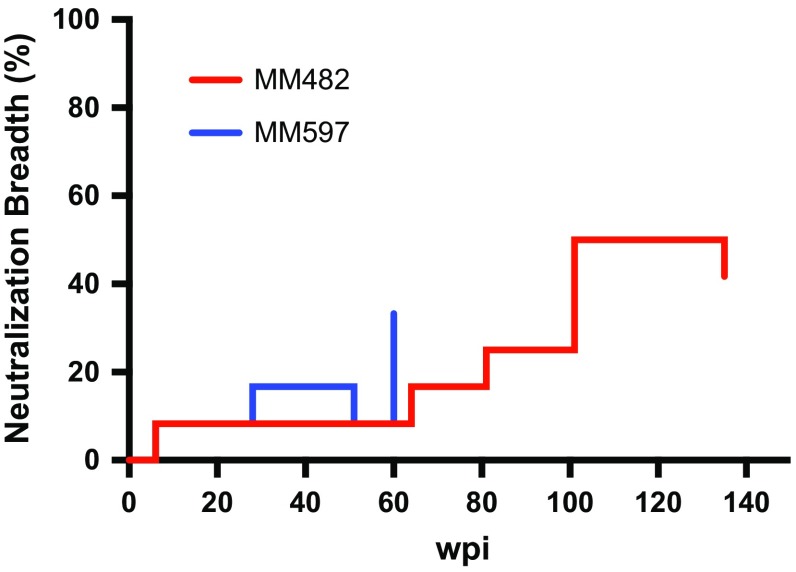
Breadth of neutralization activity in MM482 (red) and MM597 (blue). Changes in neutralization breadth against all heterologous pseudotype viruses are shown

To investigate the ability of the plasma of infected macaques to neutralize a broad spectrum of viruses, we evaluated neutralization activity against heterologous viruses. Potent neutralization of SF162, similar to that of HPP, was detected in all macaques (Table 4). In MM482 and MM597, the neutralization activity of 6535 was similar to that of HPP (ID_50_, 345 and 192, respectively) (Table 4). In MM482 and MM597, the ID_50_ value against REJO4541 was 104 and 103, respectively (Table 4). These results suggest that MM482 and MM597 have broader neutralization activity than the other five macaques, although the neutralization activity of plasma from MM482 and MM597 against tier1B and tier2 viruses was less than that of HPP in all cases (Table 4). In MM482, antibodies against infectious strains and SF162 were induced at 6 wpi (ID_50_, 755) (Table 5A). In MM597, antibodies against infectious strains were induced at 12 wpi (ID_50_, 255), and antibodies were induced against SF162 at 6 wpi (ID_50_, 300) (Table 5B). In MM482, the ID_50_ value against 6535 was 145 at 64 wpi, that against SC422661 was 111 at 81 wpi and 126 at 101 wpi, that against RHPA4259 was 596 at 101 wpi, that against QH0692 was 107 at 101 wpi and 123 at 135 wpi, and that against REJO4541 was 109 at 101 wpi and 104 at 135 wpi. At 101 and 135 wpi, two of the tier 2 panel viruses were neutralized (Table 5A and Fig. 1). In MM597, the ID_50_ value against SC422661 was 112 at 28 wpi and 105 at 60 wpi. In MM597, the ID_50_ values against 6535, REJO4541, and RHPA4259 at 60 wpi were 192, 103, and 113, respectively (Table 5B and Fig. 1). The neutralization activity of plasma from MM597 against tier 2 viruses at 60 wpi was less than that of plasma from MM482 after 101wpi. Therefore, nAbs against heterologous tier 2 virus are induced by infection with tier 1B virus after 101 wpi. It is also possible that nAbs against heterologous tier 2 viruses begin to be induced after infection with tier 2 virus. These findings suggest that antibody maturation broadens the neutralizing activity over time.

**Table 4:**
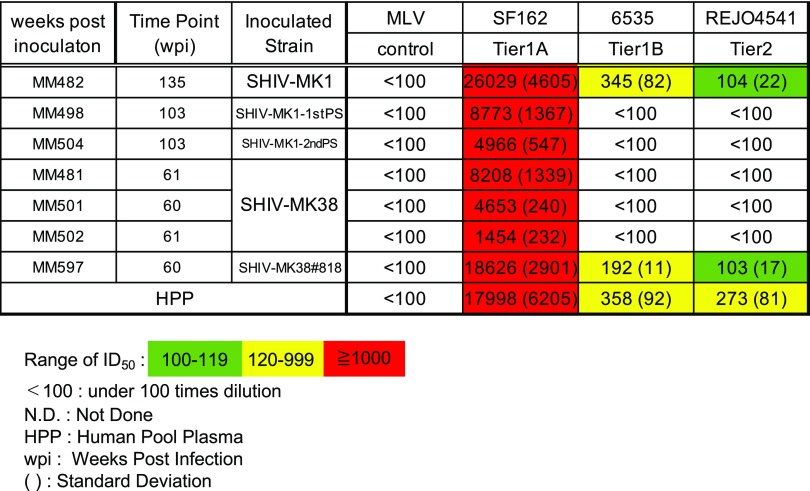
Neutralization activity against heterologous viruses

**Table 5:**
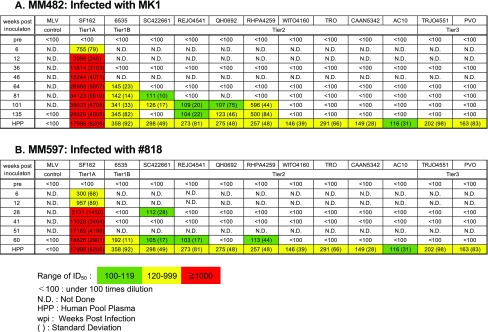
Neutralization activity and breadth against heterologous viruses over time

### Change from tier 1B to tier 2 virus in an MK1-infected macaque

**Fig. 2:**
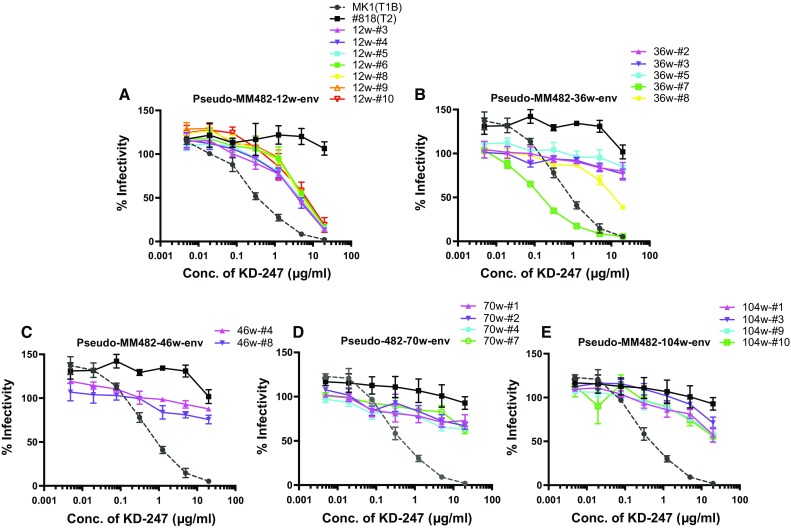
Analysis of the neutralization resistance of pseudotype viruses to HIV-1-neutralizing antibodies (nAbs) against KD247. A, pseudo-MM482-12wpi envelope. B, pseudo-MM482-36wpi envelope. C, pseudo-MM482-46wpi envelope. D, pseudo-MM482-70wpi envelope. E, pseudo-MM482-104wpi envelope. After pre-incubation with 100 TCID_50_ of each virus and KD247, TZM-bl cells were cultured for 48 h, and luciferase activity was measured. KD-247 was diluted fourfold from 20 to 0.005 µg/mL

In the body of HIV-1-infected patients, neutralizing antibodies evolve as the virus mutates [34]. Therefore, neutralizing antibodies may be produced as a tier 1B virus changes to a tier 2 virus in macaques. To confirm this, we analyzed viral resistance to neutralization over time in MM482, which had the highest and broadest neutralization activity. Pseudoviruses with the *env* gene were collected from plasma during each week of infection and evaluated for neutralization resistance using KD247, #818 (tier 2 virus), and MK 1 (tier 1 B virus). The GPGR epitope of KD 247 was preserved in all viruses from 5 to 115 wpi in the plasma of MM482. Clones 3, 4, 5, 6, 8, 9, and 10 were prepared from 12 wpi plasma. Clones 6 and 10 contained minor amino acid mutations. All of the clones showed greater resistance to neutralization than the tier 1 B MK 1 virus, but less resistance than that of the tier 2 #818 virus. At 12 wpi, all clones (3, 4, 5, 6, 8, 9, and 10) remained tier 1B (Fig. 2A). Clones 2, 3, 5, 7, and 8 were prepared from 36 wpi plasma and contained minor amino acid mutations. Because clone 7 was more susceptible to neutralization than tier 1B MK 1, it was determined to be a tier 1A virus, and because clone 8 was between MK 1 and #818, it was determined to be a tier 1B virus. Because clones 2, 3, and 5 showed neutralization resistance similar to that of #818, they were determined to be tier 2 viruses (Fig. 2B). Three of five clones were of tier 2 at 36 wpi. Eight, nine and ten clones were prepared from plasma obtained at 46, 70 and 104 wpi, respectively. The neutralization resistance of clones with minor mutations, and of those with consensus sequences, was next evaluated. At 46, 70, and 104 wpi, all clones showed neutralization resistance equivalent to that of #818 and were thus determined to be tier 2 viruses (Fig. 2C, D, and E). These results suggest that tier 2 virus appeared at 36 wpi, and further that only tier 2 viruses proliferated after 46 wpi in MM482, which was infected with tier 1B MK1.

### Mutations related to neutralization resistance and induction of broadly neutralizing antibodies

**Fig. 3:**
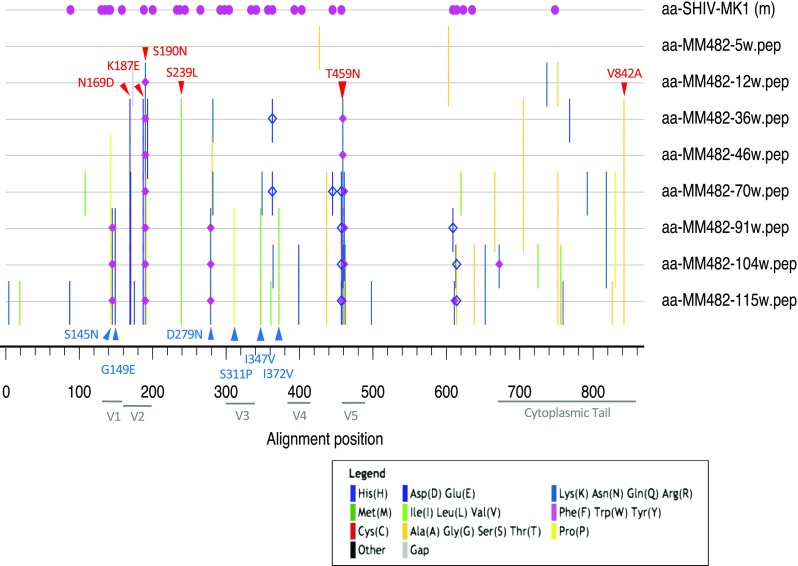
Amino acid mutations post-inoculation in the plasma of MM482 infected with MK1. The original sequence is the SHIV-MK1 sequence. Pink circle, potential N-linked glycosylation site in the master. Pink diamond, potential N-linked glycosylation sites gained in the query compared to the master. Blue open diamond, potential N-linked glycosylation sites lost in the query compared to the master. Red, common mutations after 36 wpi. Blue, common mutations from 91 to 115 wpi. Minor mutations in the consensus sequence are not shown

To identify mutations related to neutralization resistance and induction of broadly neutralizing antibodies, a mutation analysis of *env* was performed. Direct sequencing showed a consensus sequence lacking minor mutations (Fig. 3). The minor mutations detected in the neutralization-susceptible clones 7 and 8 at 36 wpi were not included. First, common mutations were found from 36 wpi (when neutralization-resistant tier 2 virus appeared) to 115 wpi. These comprised N169D, K187E, S190N in the V2 region, S239 in the C2 region, T459N in the V5 region (T459D at 91 wpi), and V842A in the cytoplasmic tail (Fig. 3, red font). S190N and T459N gained more potential N-linked glycosylation sites compared to MK1. Next, common mutations were found from 91 to 115 wpi; the former was the timepoint at which the maximum neutralizing activity against heterologous viruses was detected. We found the mutations S145N and G149E in the V1 region, D279N in the C2 region, S311P in the V3 region, and I347V and I372V in the C3 region (Fig. 3, blue font). S145N and D279N gained potential N-linked glycosylation sites compared to MK1. These findings imply that these mutations are related to neutralization resistance and induction of broadly neutralizing antibodies.

## Discussion

The SHIV strains MK1, MK38, and #818, which were derived from SHIV-89.6, are CCR5-tropic and have different levels of resistance to neutralization (tier 1B and 2) [51, 52]. These viruses are genetically similar to SHIV-89.6 P [50], which is widely used in vaccine development. In this study, we developed a rhesus macaque model of induction of anti-HIV-1 nAbs.

In MM597, nAbs against parental-lineage tier 2 viruses were rapidly induced, and nAbs against heterologous tier 2 viruses were beginning to be induced (Tables 3 and 5; Fig. 1). In HIV-1-infected patients, self- or type-specific Ab responses develop first, followed by Abs with increased affinity and neutralization activity against autologous viruses [34]. Indeed, neutralization activity against parental-lineage virus increased in rhesus macaques infected with CCR5-tropic SHIV. Moreover, in HIV-1-infected patients with nAbs against autologous virus, escape mutants are generated in the virus, and the *env* sequence diversity increases. Subsequently, the host humoral immune response results in production of nAbs with increased affinity. After a number of years, some patients produce antibodies that target one or more shared epitopes, resulting in cross-reactivity with heterologous strains. This leads to induction of broadly neutralizing antibodies with activity against diverse tier 2 viruses [34]. Therefore, #818-infected rhesus macaques mimic nAb induction in HIV-1-infected patients and may be used to evaluate the induction of tier 2 nAbs.

KS661 was susceptible to neutralization (tier 1B). Induction of Abs in macaques infected with KS661 inhibits viral replication; however, MK38 became resistant to neutralization (tier 2) [52]. MK38 and #818 established persistent infections despite nAb production (Tables 2 and 4), possibly due to the emergence of neutralization-escape mutations or to resistance to nAbs due to the three-dimensional structure of the virus. Indeed, when the co-receptor changes from CXCR4 to CCR5, the resulting decrease in the net positive charge of V3 reduces its surface exposure, resulting in immunological escape from nAbs [52, 59].

In MM482, the N169D, K187E, S190N, S239, T459N (T459D at 91 wpi), and V842A mutations were detected at 36 wpi. Because the tier 2 #818 virus has three mutations in the V2 region (N169D, K187E, and S190N) (Supplemental Figure), the above-mentioned six mutations likely contribute to neutralization resistance.

nAbs against tier 2 parental-lineage and heterologous viruses can be induced by infection with tier 1B virus (Tables 2, 3, and 5; Fig. 1). In HIV-1-infected patients, neutralization results from viral mutations [34]. We analyzed viral resistance to neutralization over time in MM482. In MM482 infected with tier 1B MK 1 virus, the virus mutated from tier 1B to tier 2 at 36 wpi. In MM482, nAbs against the tier 2 #818 virus were detected at 64 wpi (Table 3A), and against two of the heterologous tier 2 viruses in the panel after 101 wpi (Table 5A). In MM597 infected with the tier 2 #818 virus, nAbs against three strains of the heterologous tier 2 virus panel were beginning to be detected at 60 wpi, although their ID_50_ values were low (Table 5). These results imply that induction of broadly neutralizing antibodies occurs more than 60 weeks after infection with neutralization-resistant virus.

In MM482, the S145N, G149E, D279N, S311P, I347V and I372V mutations were detected from 91 wpi to 115 wpi. Since the maximum neutralizing activity against heterologous viruses was detected at this time, six mutations may contribute to the induction of broadly neutralizing antibodies. Analysis of the epitopes targeted by the nAbs induced by MM482 is needed, together with verification that the mutations detected after 91 wpi are important for induction of broadly neutralizing antibodies. It is possible that induction of broadly neutralizing antibodies can be accelerated by a viral antigen with mutations related to neutralization resistance and induction of broadly neutralizing antibodies.

Based on our observations, #818-infected rhesus macaques may be useful models for the induction of tier 2 nAbs. In addition, MK1 (tier 1B)-infected rhesus macaques will enable analysis of the neutralization resistance of viruses that induce tier 2 nAbs and the antigen needed to induce broadly neutralizing antibodies. Finally, these animal models will facilitate the development of HIV-1 vaccines.

## Electronic supplementary material

Below is the link to the electronic supplementary material.

Supplementary material 1 (PDF 58 kb)
