# Supplementary material for: Induction of neutralizing antibodies against tier 2 human immunodeficiency virus 1 in rhesus macaques infected with tier 1B simian/human immunodeficiency virus
Source: Arch Virol. 2019 Feb 28;164(5):1297–308. doi: 10.1007/s00705-019-04173-5 (PMC6469619; doi:10.1007/s00705-019-04173-5)

Supplemental Figure. Amino acid mutations in the plasma of MM482 infected with MK1 virus. Original sequence, SHIV-MK1 sequence.

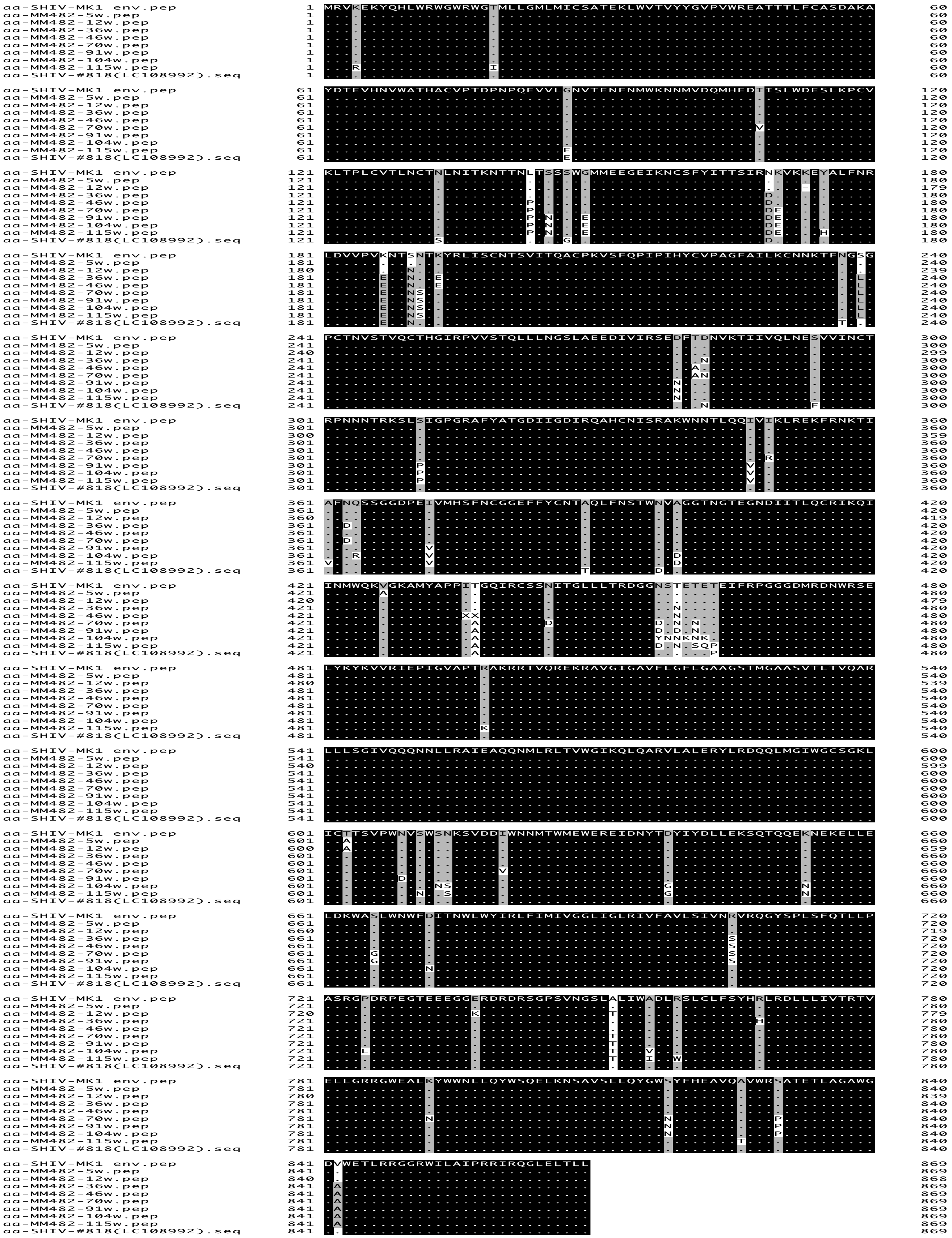

Supplement: Supplementary file 1 — Supplementary material 1 (PDF 58 kb) [file 705_2019_4173_MOESM1_ESM.pdf]
